# Effects of physical activity levels on characteristic pain in temporomandibular dysfunctions: a cross-sectional study

**DOI:** 10.1186/s13005-024-00407-3

**Published:** 2024-01-18

**Authors:** Youngwoo Chun, Jung Hwan Jo, Ji Woon Park

**Affiliations:** 1 Department of Oral Medicine, Seoul National University Dental Hospital, 101, Daehak-ro, Jongno-gu, Seoul, 03080 South Korea; 2 Department of Oral Medicine and Oral Diagnosis, School of Dentistry, Seoul National University, 101 Daehak-ro, Jongno-gu, Seoul, 03080 South Korea; 3 Dental Research Institute, Seoul National University, 101 Daehak-ro, Jongno-gu, Seoul, 03080 South Korea

**Keywords:** Temporomandibular disorders, Physical activity, Actigraphy, Pain, Comorbidity

## Abstract

**Background:**

Physical activity is known to influence the symptoms of a variety of pain disorders including fibromyalgia and osteoarthritis although the underlying mechanism is not fully understood. In spite of the high prevalence of temporomandibular disorders (TMD), no previous study has objectively evaluated the relationship between TMD and general physical activity. This study aims to investigate the influence of physical activity on pain and disability from TMD, considering various confounders including sleep, systemic inflammation, psychosocial disturbances, and widespread pain.

**Methods:**

This observational cross-sectional study is based on consecutive samples of 100 TMD patients (22 with high pain disability and 78 with low pain disability level). Physical activity levels were assessed with actigraph. Level of pain and disability were evaluated using the Graded Chronic Pain Scale. Hematologic examinations including inflammatory biomarkers were assessed and comorbidities were investigated with validated questionnaires. Differences were analyzed according to disability level.

**Results:**

Patients with high disability level spent significantly more time doing both moderate (*p* = 0.033) and vigorous (*p* = 0.039) level physical activity. Light physical activity, on the other hand, was associated with low disability but the difference did not reach statistical significance. Time spent in light physical activity was significantly associated with high levels of pain and disability (*p* = 0.026, β = −0.001) and time spent in vigorous physical activity had significant predictive power (cutoff value 2.5 min per week, AUC 0.643, *p* = 0.041). Scores of the Jaw Function Limitation Score-20 (*p* = 0.001), present McGill Pain Score (*p* = 0.010), and number of people potentially diagnosed with fibromyalgia (*p* = 0.033) were significantly higher in the high disability group.

**Conclusions:**

Moderate or vigorous physical activity is associated with worse TMD symptoms while light physical activity may be beneficial. Further research related to the amount and frequency of physical activity is necessary to establish clinical guidelines for TMD.

**Trial registration:**

clinical trial registration of the Clinical Research Information Service of Republic of Korea (number KCT0007107).

## Introduction

Temporomandibular disorders (TMD) is characterized by pain and functional problems involving the temporomandibular joint (TMJ) and masticatory musculature, which is the second most common cause of nonodontogenic pain in the orofacial area with a prevalence of 5–12% of the adult population [1]. Many factors including genetic, anatomic, hormonal, sleep quality, and psychosocial conditions are known to be involved in its initiation and exacerbation [2, 3].

Physical activity defined as “any bodily movement produced by skeletal muscles that requires energy expenditure” by World Health Organization (WHO) [4] is known to influence the symptoms of a variety of pain disorders. Fibromyalgia patients reported significantly lower perceived pain levels when exercise was implemented [5]. Similarly, it was reported that physical activity can relieve pain and enhance joint function of patients with knee or hip osteoarthritis [6]. On the other hand, high intensity exercise was correlated to increased pain of patients with fibromyalgia and low back pain [5, 7]. The underlying mechanism of such interactions are not fully understood and specific guidelines regarding physical activity intensity and duration related to distinct disease entities are yet to be provided.

In spite of its high prevalence and impact on quality of life with prolonged symptoms, no previous study has objectively evaluated the relationship between TMD and general physical activity while considering established confounders such as psychological problems and sleep. Sleep quantity and quality have major influences on pain characteristics, and the proper management of related issues are known to result in favorable treatment response [8–10]. Physical activity, sleep, and pain show a strong interrelationship with one affecting the one through overlapping mechanisms [11–13]. One common underlying factor may be the presence of nonspecific inflammation that is known to be involved in all three physiological states. The association between certain hematological markers indicating systemic inflammation and pain levels have been reported, however the results were inconsistent. High-intensity exercise may produce inflammatory mediators, which in turn could increase pain levels [14]. Nevertheless, another study reported that moderate to vigorous physical activity was significantly correlated with lower inflammatory biomarker levels such as high sensitivity C-reactive protein (CRP) [15]. Another confounder, psychologic disturbances including anxiety and depression are also crucial when interpreting the effect of physical activity but often omitted in analysis.

Therefore, the objective of this study was to investigate the correlation of physical activity and pain in a well-defined group of TMD patients, taking into account various confounders including sleep, systemic inflammation, and psychosocial disturbances. Also, the value of physical activity level as a predictive index of TMD severity was analyzed to provide a guideline in TMD.

## **Patients and methods**

### Study design

The protocol of this cross-sectional study can be found in a previous paper [16]. Those who visited the Orofacial Pain Clinic of Seoul National University Dental Hospital with the chief complaint of discomfort of the TMJ area were recruited from May, 2021 to February 2022. To prevent selection bias, patients were recruited sequentially in the order they arrived. The Institutional Review Board (IRB) of the same hospital (CRI21007) approved the study protocol and written informed consent was obtained from all participants. The study was registered in the Clinical Research Information Service of Republic of Korea (KCT0007107). All procedures complied with multiple ethical standards of the institutional research committee, the Helsinki declaration in 1964, and its later amendments or other equivalent ethical standards.

### Subjects

**Fig. 1:**
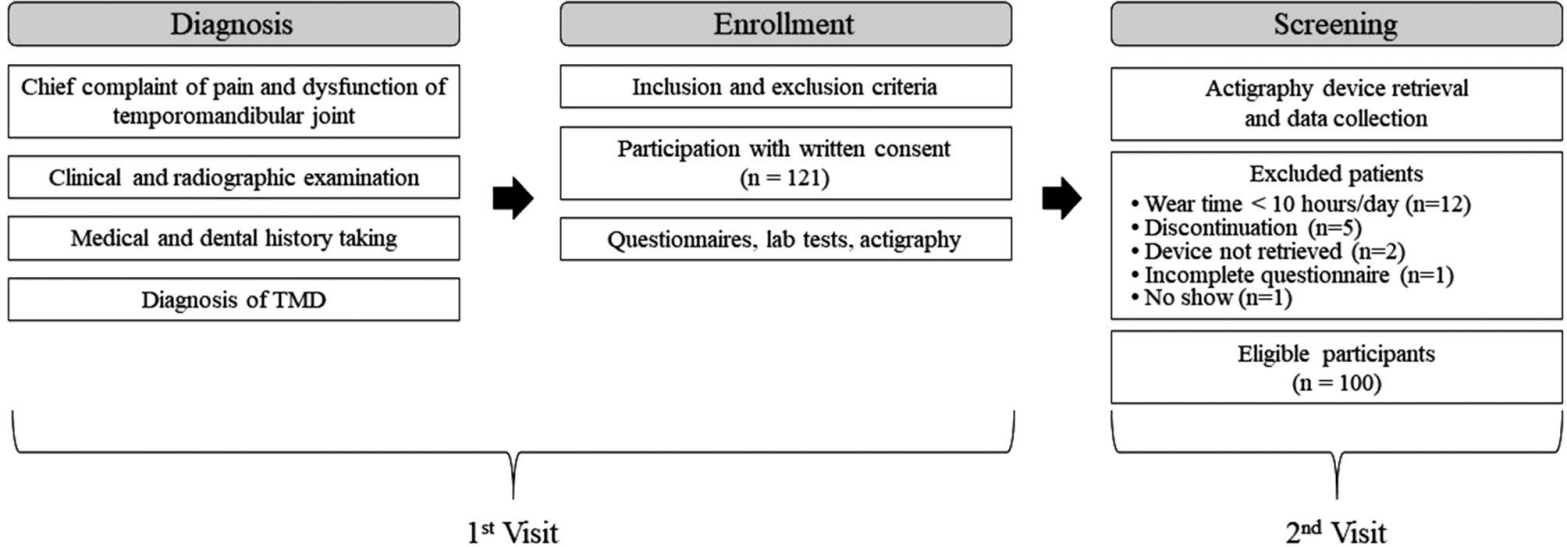
Flowchart of the study procedure

Participants with Korean nationality who were ≥ 18 years old were included. TMD was diagnosed according to the Diagnostic Criteria for TMD (DC/TMD) [17]. Patients with previously diagnosed systemic musculoskeletal disorders, uncontrolled endocrine, liver, or kidney disorder, autoimmune disease, trauma within the last 6 months, psychiatric disorder that may affect the study, and primary sleep disorder diagnosis were excluded. A total of 121 patients provided written consent. The flowchart of the whole study process is illustrated in Fig. 1. The participants were grouped according to pain disability level of the Graded Chronic Pain Scale (GCPS) of DC/TMD Axis II as low (low disability-low intensity pain and low disability-high intensity pain) and high (high disability-moderately limiting pain and high disability-severely limiting pain) disability groups for statistical analysis [18].

### Clinical assessment

On the first visit, hematologic examinations including complete blood cell count (CBC), erythrocyte sedimentation rate (ESR), and high sensitivity CRP, rheumatoid factor (RF), fluorescent antinuclear antibody (FANA), and anti-cyclic citrullinated peptide (Anti-CCP) were implemented. Several inflammatory markers showing significant correlation with disease activity and mortality, such as platelet-to-lymphocyte ratio (PLR), neutrophil-lymphocyte ratio (NLR), and derived neutrophil-lymphocyte ratio (dNLR) were calculated [19–22].

Confounding factors were investigated with well-known validated questionnaires applied in various previous studies, which are summarized in Table 1. Validated Korean versions were completed on the first visit. Also, questionnaires of the DC/TMD axis II including Jaw Function Limitation Scale-20 (JFLS-20), Oral Behavior Checklist (OBC), General Anxiety Disorder-7 (GAD-7), Patient Health Questionnaire-9 (PHQ-9), and Patient Health Questionnaire-15 (PHQ-15) were implied [18].

**Table 1:** List of structured questionnaires to measure comorbidities

Section	Questionnaire	Description	Score range
Physical activity	International physical activity questionnaire [23]	Self-reported physical activity level	1–3
	Tampa Scale of Kinesiophobia for Temporomandibular Disorders (TSK-TMD) [24]	Fear of movement and activity	18–72
General health	Short Form 36 (SF-36) [25]	Difficulties in various activities	0–100
	Composite Autonomic Symptom Score 31 (COMPASS 31) [26]	Autonomic symptoms	0-100
	Short form McGill Pain Questionnaire (MPQ) [27]	Quality and intensity of pain	0–45
Sleep disturbance and fatigue	Pittsburgh Sleep Quality Index (PSQI) [28]	Quality of sleep	0–21
	Epworth Sleepiness Scale (ESS) [29]	Daytime sleepiness	0–24
	Fatigue Assessment Instrument (FAI) [30]	Fatigue and related medical disorders	1–7
	Insomnia Severity Index (ISI) [31]	Severity of insomnia	0–28
	Morningness-eveningness questionnaire (MEQ) [32]	Preference of when to start sleep or wake up	16–86
Widespread pain	Symptom Severity (SS) scale	Widespread body pain and centralized pain characteristics	0–12/0–19
	Widespread Pain Index (WPI) [33]		
	Fibromyalgia Impact Questionnaire (FIQ) [34]	Pain and functional impact of fibromyalgia	0-100
Psychologic disturbance	Symptom Checklist-90-Revised (SCL-90-R) [35]	Psychological problems	
	Beck Depression Index (BDI) [36]	Emotional and behavioral depression	0–63
	Beck Anxiety Index (BAI) [37]	Emotional and physical anxiety	0–63
	Pain Catastrophizing Scale (PCS) [38]	Negative thoughts and emotions about pain	0–52
	Central Sensitization Inventory (CSI) [39]	Symptoms related to central sensitization	0-100
	Pennebaker Index of Limbic Languidness (PILL) [40]	Tendency to notice physical symptoms and sensations	0-216
	Perceived Stress Scale (PSS) [41]	Nonspecific perceived stress	0–40

#### Physical activity assessment

**Fig. 2:**
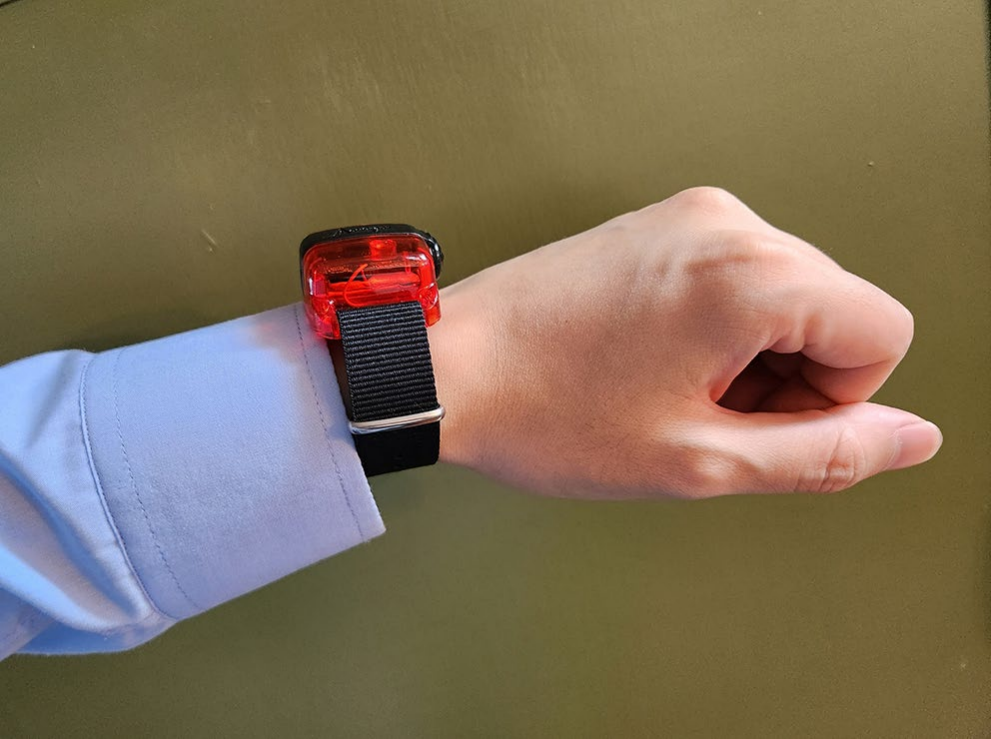
Actigraph wGT3X-BT (ActiGraph, Florida, USA)

Actigraph wGT3X-BT (Fig. 2) with proven reliability for assessing continuous physical activity data was used to objectively monitor the periods of sleep, rest, and activity [42–45]. Participants wore the device 24 h a day for 7 consecutive days starting from the day of the first visit. Recording ended automatically after this period. It was worn on the wrist, either dominating or non-dominating side. An epoch was 60 s, and the bout setting was customized to count bouts regardless of length according to the most recent WHO recommendation [46]. The device was collected on the next visit, and data were downloaded using ActiLife v6.13.4 (ActiGraph, Florida, USA). Since wear time of more than 10 h a day is considered as compliant according to Choi’s wear time validation, only participants who wore the device for an average of 10 h or more per day were included [47]. Choi’s wear time validation has been proven to be reliable and superior to Troiano technique, accurately reflecting actual wear time with less error for wrist-worn devices in a free-living setting [48, 49]. Cutoff values of 232, 4,514, and 15,044 vector magnitude counts were applied for light, moderate, and vigorous activity, respectively [50].

### Statistical analysis

Shapiro-Wilk test was used to test normality of data with following methods selected accordingly. Differences in outcomes between groups were analyzed with Student’s t-test or Mann–Whitney U test for continuous variables and chi-square test or Fisher’s test for discrete variables. After evaluation of multicollinearity, a limited number of variables were selected to enter logistic regression analysis based on presence of significance in independent comparison analysis and also clinical relevance. The eight variables chosen displayed a correlation coefficient of less than 0.8 with respect to each other. Additionally, collinearity diagnostic analysis was conducted individually for each of the 8 variables, confirming variance inflation factor values below 5 and tolerance values above 0.2. Logistic regression with backward elimination was used to investigate actigraphy indices associated with clinical outcomes according to groups. Receiver operating characteristic (ROC) curve and area under the curve (AUC) were analyzed to evaluate the predictive ability of physical activity for TMD disability level. Complete case analysis, a method which involves excluding any observations with missing data was used. All data were analyzed using IBM Corp. released 2019. IBM SPSS Statistics for Windows, Version 26.0. (Armonk, NY: IBM Corp). The significance level was set at 0.05.

## Results

### Clinical TMD indices

As shown in Table 2, there was no significant difference in age, body mass index (BMI), and sex between the two groups. Those in the high disability group reported significantly more limitation in jaw function (*p* = 0.001) and higher pain intensity based on MPQ (*p* = 0.010) and 0–10 numeric rating scale (NRS, *p* = 0.021). All other clinical indices consistently reflected a more severe state of TMD symptoms in the high disability group however, the difference was not statistically significant.

**Table 2:** Clinical characteristics according to different disability level groups

	Low (*n* = 78)	High (*n* = 22)	P-value
Age^††^	29.00 (24.00, 45.25)	27.00 (24.00, 35.75)	0.386
Sex (Female)^‡‡^	79.49% (62/78)	86.36% (19/22)	0.555
BMI^†^	22.39 (3.31)	21.94 (3.23)	0.578
BMI ≥ 25^‡‡^	17.95% (14/78)	13.64% (3/22)	0.757
JFLS-20^†^	1.65 (1.33)	2.83 (1.75)	0.001*
OBC^†^	17.17 (10.47)	21.55 (9.91)	0.099
OBC > 20^‡^	32.47% (25/77)	54.55% (12/22)	0.080
CMO (mm)^†^	40.01 (9.88)	37.55 (11.47)	0.296
MMO (mm)^†^	43.08 (8.92)	43.32 (10.15)	0.956
Masticatory muscle palpation (number of positive sites)^††^	1.00 (0.00, 5.50)	1 (0.00, 9.75)	0.262
Cervical muscle palpation (number of positive sites)^††^	0.00 (0.00, 1.00)	0.00 (0.00, 0.00)	0.583
Capsule palpation (number of positive sites)^††^	0.00 (0.00, 1.00)	0 (0.00, 2.00)	0.214
Pain on mouth opening^‡^	46.15% (36/78)	68.18% (15/22)	0.091
Pain on lateral movement^‡^	34.62% (27/78)	40.91% (9/22)	0.620
Pain on protrusive movement^‡^	25.64% (20/78)	27.27% (6/22)	1.000
Tooth attrition^‡‡^	16.88% (13/77)	28.57% (6/21)	0.230
Tongue ridging^‡^	43.42% (33/76)	57.14% (12/21)	0.326
Mucosal ridging^‡^	59.21% (45/76)	80.95% (17/21)	0.077
**MPQ**			
Total^††^	5.00 (2.00, 8.00)	7.00 (4.25, 10.75)	0.154
Average^†^	0.34 (0.25)	0.43 (0.19)	0.126
Now^††^	1.00 (1.00, 2.00)	2.00 (2.00, 2.00)	0.010*
Initial NRS^†^	3.78 (2.27)	5.05 (1.80)	0.021*
DJD^‡‡^	85.90% (67/78)	81.82% (18/22)	0.736

BMI: Body mass index, JFLS-20: Jaw function limitation scale-20, OBC: Oral behavior checklist, CMO: Comfortable mouth opening, MMO: Maximum mouth opening, MPQ: McGill pain questionnaire, NRS: Numeric rating scale, DJD: Degenerative joint disease

^†^Differences between groups were tested with independent t-test: mean (standard deviation)

^††^Differences between groups were tested with Mann-Whitney test: median (lower quartile, upper quartile)

^‡^Differences between groups were tested with chi-square test: number of subjects or positive palpation sites (%)

^‡‡^Differences between groups were tested with Fisher’s exact test: number of subjects or positive palpation sites (%)

^*^Significant difference, *p* < 0.05

### Physical activity and sleep indices

Unlike subjective levels of physical activity based on IPAQ which did not show a difference, certain objective measurements were significantly different between groups as shown in Table 3. High disability TMD patients participated in more moderate (*p* = 0.033) and vigorous (*p* = 0.039) physical activities. Those with low disability spent more time doing light physical activity although the difference was not significant from the high disability group.

**Table 3:** Physical activity and sleep indices according to different disability level groups

	Low (*n* = 78)	High (*n* = 22)	P-value
**IPAQ**			
Categorical^‡^	24.36% (19/78)	23.81% (5/21)	1.000
Continuous^††^	2006.25 (717.75, 4129.50)	2112.00 (717.75, 5482.50)	0.635
Average kcals per day^†^	1226.12 (471.73)	1173.86 (508.32)	0.656
METs^†^	2.15 (0.22)	2.11 (0.24)	0.422
**Physical activity per week (min)**			
Light^†^	3790.28 (726.11)	3462.86 (965.89)	0.090
Moderate^†^	1185.19 (556.88)	1203.86 (571.11)	0.033*
Vigorous^††^	3.00 (1.00, 9.00)	7.00 (3.00, 11.00)	0.039*
Total MVPA^†^	1195.62 (561.02)	1219.27 (587.09)	0.035*
Time in sedentary bouts per day^†^	313.20 (87.95)	324.19 (69.76)	0.594
Time in sedentary breaks per day^†^	1036.40 (103.12)	1030.42 (77.53)	0.803
Sedentary bouts per day^†^	14.64 (4.06)	15.23 (3.90)	0.553
Sedentary bouts average length^†^	21.44 (1.88)	21.80 (2.73)	0.582
Sedentary breaks average length^††^	74.10 (60.75, 84.70)	66.15 (56.80, 75.53)	0.241
Axis 1 CPM^†^	864.64 (272.28)	847.50 (265.39)	0.796
Axis 2 CPM^†^	888.85 (260.35)	896.08 (257.81)	0.860
Axis 3 CPM^†^	981.29 (319.55)	988.28 (308.45)	0.928
**Vector magnitude**			
Average counts^†^	1619.22 (470.77)	1629.19 (482.40)	0.931
CPM^†^	1581.05 (487.66)	1582.45 (475.18)	0.991
Step counts per day^†^	10383.96 (3080.31)	10225.73 (3578.22)	0.840
Lux average counts^††^	25.30 (7.18, 55.40)	16.55 (4.65, 50.05)	0.446
Minutes in bed^††^	449.81 (417.89, 513.33)	480.92 (424.01, 529.54)	0.318
Minutes in bed < 420^‡^	26.92% (21/78)	22.73% (5/22)	0.695
Total Sleep Time (TST)^††^	391.86 (362.96, 463.44)	424.74 (388.32, 458.64)	0.191
Total Sleep Time (TST) < 420^‡^	60.26% (47/78)	50.00% (11/22)	0.466
Wake After Sleep Onset (WASO)^††^	52.46 (42.32, 66.48)	48.67 (41.13, 59.33)	0.621
Sleep efficiency^††^	88.41 (85.57, 90.86)	90.22 (87.85, 91.11)	0.190
Sleep fragmentation index^†^	25.13 (7.20)	23.00 (4.81)	0.198
Movement index^†^	13.04 (3.21)	11.96 (2.66)	0.156
Fragmentation index^†^	12.09 (4.88)	11.03 (3.56)	0.352

IPAQ: International physical activity questionnaire, METs: Metabolic equivalent of tasks, MVPA: Moderate-to-vigorous physical activity, CPM: Counts per minute

^†^Differences between groups were tested with independent t-test: mean (standard deviation)

^††^Differences between groups were tested with Mann-Whitney test: median (lower quartile, upper quartile)

^‡^Differences between groups were tested with chi-square test: number of subjects or positive palpation sites (%)

^‡‡^Differences between groups were tested with Fisher’s exact test: number of subjects or positive palpation sites (%)

^*^Significant difference, *p* < 0.05

There was no significant difference in sleep indices between the two groups.

### Comorbidity levels

Table 4 shows comorbidity levels of both groups. Although the results of FIQ had no significant difference, the number of people diagnosed with fibromyalgia based on the SS scale and WPI according to the 2016 American College of Rheumatology diagnostic criteria was higher in the high disability group (*p* = 0.033).

**Table 4:** Comorbidity levels according to different disability level groups

	Low (*n* = 78)	High (*n* = 22)	P-value
ESS^†^	6.77 (4.49)	6.23 (3.38)	0.544
ESS > 10^‡‡^	18.18% (14/77)	13.64% (3/22)	0.756
PSQI^†^	7.90 (3.41)	7.91 (2.73)	0.987
PSQI ≥ 5^‡^	85.71% (66/77)	90.91% (20/22)	0.727
FAI^†^	4.14 (1.01)	4.43 (0.71)	0.207
FIQ^†^	29.39 (19.54)	31.78 (16.26)	0.605
FM diagnosis^‡‡^	1.30% (1/77)	13.64% (3/22)	0.033*
WPI^††^	3.00 (2.00, 6.00)	4.00 (2.00, 5.00)	0.809
SS^†^	4.71 (2.24)	5.05 (2.25)	0.546
**COMPASS 31**			
Orthostatic intolerance^††^	20.00 (8.00, 28.00)	22.00 (8.00, 28.00)	0.920
Vasomotor^††^	1.67 (1.67, 1.67)	1.67 (1.67, 1.67)	0.871
Secretomotor^†^	18.34 (3.19)	19.29 (2.69)	0.208
Gastrointestinal^††^	15.18 (10.71, 16.96)	12.95 (9.15, 16.74)	0.247
Bladder^††^	3.33 (3.33, 4.44)	3.33 (3.33, 3.33)	0.296
Pupillomotor^††^	2.67 (1.00, 3.33)	1.00 (1.00, 3.25)	0.210
Total^†^	60.85 (15.26)	59.58 (12.11)	0.723
CSI^†^	30.38 (17.59)	31.68 (15.43)	0.756
CSI ≥ 30^‡^	41.56% (38/77)	50% (11/22)	1.000
BDI^†^	8.45 (8.14)	9.41 (7.01)	0.622
BDI ≥ 30^‡^	1.30% (1/77)	0.00% (0/22)	1.000
BAI^††^	4.00 (2.00, 11.00)	5.00 (2.00, 10.25)	0.847
BAI ≥ 26^‡‡^	3.90% (3/77)	4.55% (1/22)	1.000
GAD-7^†^	4.03 (4.89)	4.09 (3.30)	0.982
PILL^†^	52.71 (31.98)	57.36 (31.87)	0.553
PILL > 84^‡‡^	18.18% (14/77)	13.64% (3/22)	0.756
PSS^†^	16.10 (7.37)	14.68 (7.05)	0.427
PSS > 13^‡^	62.34% (48/77)	50% (11/22)	0.332
TSK-TMD^†^	26.76 (6.71)	29.59 (5.01)	0.082
TSK-TMD ≥ 23^‡‡^	78.21% (61/78)	86.36% (19/22)	0.551
**SF-36**			
Physical functioning^††^	95.00 (80.00, 100.00)	95.00 (76.25, 100.00)	0.973
Role physical^††^	100.00 (50.00, 100.00)	75 (31.25, 100.00)	0.080
Role emotional^††^	100.00 (66.67, 100.00)	66.67 (41.67, 100.00)	0.073
Energy/fatigue^†^	49.68 (20.39)	46.82 (19.75)	0.565
Emotional well-being^†^	67.90 (20.02)	66.55 (14.46)	0.924
Social functioning^††^	87.50 (67.50, 100.00)	87.50 (69.38, 90.00)	0.646
Pain^†^	64.51 (21.98)	56.59 (19.81)	0.196
General health^†^	53.44 (22.71)	54.09 (16.56)	0.804
PHQ-9^†^	4.41 (4.95)	5.50 (4.24)	0.354
PHQ-9 ≥ 20^‡‡^	1.28% (1/78)	0.00% (0/22)	1.000
PHQ-15^†^	5.76 (4.61)	6.45 (3.96)	0.524
PHQ-15 ≥ 15^‡‡^	6.41% (5/78)	4.55% (1/22)	1.000
Headache^‡^	41.56% (32/77)	63.64% (14/22)	0.408

ESS: Epworth sleepiness scale, PSQI: Pittsburgh sleep quality index, FAI: Fatigue assessment instrument, FIQ: Fibromyalgia impact questionnaire, FM: Fibromyalgia, WPI: Widespread pain index, SS: Symptom severity scale, COMPASS 31: Composite autonomic symptom score 31, CSI: Central sensitization index, BDI: Beck depression index, BAI: Beck anxiety index, GAD-7: General anxiety disorder-7, PILL: Pennebaker index of limbic languidness, PSS: Perceived stress scale, TSK-TMD: Tampa scale of kinesiophobia for TMD, SF-36: Short form-36, PHQ-9: Patient health questionnaire-9, PHQ-15: Patient health questionnaire-15

^†^Differences between groups were tested with independent t-test: mean (standard deviation)

^††^Differences between groups were tested with Mann-Whitney test: median (lower quartile, upper quartile)

^‡^Differences between groups were tested with chi-square test: number of subjects or positive palpation sites (%)

^‡‡^Differences between groups were tested with Fisher’s exact test: number of subjects or positive palpation sites (%)

^*^Significant difference, *p* < 0.05

### Hematologic indices

As shown in Table 5, none of the examined indices was significantly different between the two groups. Indices such as PLR, NLR, dNLR, SII, ESR, and hs-CRP were higher while LMR was lower in the high disability group, which reflects the possibility of systemic inflammation although the difference was not statistically significant.

**Table 5:** Hematologic indices of systemic inflammation according to different disability level groups

	Low (*n* = 77)	High (*n* = 22)	P-value
WBC^†^	5.83 (1.42)	6.02 (1.39)	0.584
WBC group (≥ 10)^‡‡^	1.30% (1/77)	0.00% (0/22)	1.000
RBC^†^	4.51 (0.37)	4.43 (0.34)	0.355
RBC group (≥ 5.40)^‡‡^	6.49% (5/77)	9.09% (2/22)	0.650
Hgb^††^	13.70 (12.80, 14.30)	13.60 (12.65, 14.05)	0.426
Hgb group (< 12)^‡‡^	2.60% (2/77)	4.55% (1/22)	0.534
Hct^†^	40.71 (3.11)	39.96 (2.74)	0.315
Hct group (≤ 36)^‡‡^	3.90% (3/77)	9.09% (2/22)	0.307
Platelet^†^	262.90 (47.60)	269.32 (51.68)	0.589
PLR^††^	82.87 (30.38)	86.88 (32.68)	0.749
PLR group (≥ 142.76 (F), ≥ 122.73 (M))^‡‡^	3.90% (3/77)	4.55% (1/22)	1.000
NLR^††^	1.13 (0.76)	1.25 (0.83)	0.614
NLR group (≥ 1.662 (F), ≥ 1.634 (M))^‡‡^	15.58% (12/77)	27.27% (6/22)	0.222
dNLR^†^	1.40 (0.53)	1.47 (0.62)	0.601
LMR^††^	5.10 (3.93, 6.26)	4.83 (3.84, 5.68)	0.711
LMR group (≤ 5.598 (F), ≤ 5.048 (M))^‡^	33.77% (26/77)	31.82% (7/22)	1.000
SII^††^	249.58 (171.78, 356.45)	255.39 (181.97, 399.05)	0.668
ESR (mm/h)^††^	5.00 (2.00, 10.00)	9.00 (4.25, 14.75)	0.440
CRP (mg/L)^††^	0.04 (0.02, 0.08)	0.05 (0.04, 0.08)	0.354
RF^‡‡^	1.30% (1/76)	4.50% (1/22)	0.400
FANA^‡^	29.90% (23/77)	18.20% (4/22)	0.416
Anti-CCP^‡‡^	3.90% (3/77)	0.00% (0/22)	1.000
Total protein (g/dL)^††^	7.40 (7.1, 7.7)	7.30 (7.0, 7.58)	0.660

WBC: White blood cell, RBC: Red blood cell, Hgb: Hemoglobin, Hct: Hematocrit, PLR: Platelet-to-lymphocyte ratio, NLR: Neutrophil-lymphocyte ratio, dNLR: derived neutrophil-lymphocyte ratio, LMR: Lymphocyte-monocyte ratio, SII: Systemic inflammatory index, ESR: Erythrocyte sedimentation rate, CRP: C-reactive protein, RF: Rheumatoid factor, FANA: Fluorescent antinuclear antibody, anti-CCP: anti-cyclic citrullinated peptide

^†^Differences between groups were tested with independent t-test: mean (standard deviation)

^††^Differences between groups were tested with Mann-Whitney test: median (lower quartile, upper quartile)

^‡^Differences between groups were tested with chi-square test: number of subjects or positive palpation sites (%)

^‡‡^Differences between groups were tested with Fisher’s exact test: number of subjects or positive palpation sites (%)

^*^Significant difference, *p* < 0.05

### Physical activity indices indicating high disability TMD

Logistic regression analysis results are shown in Table 6. As a result of analyzing VIF and tolerance for all variables, all values were less than 5 and exceeded 0.2, respectively. All values derived from Pearson bivariate correlation analysis were less than 0.8. No significant multicollinearity between any variables was found. Time spent in light physical activity (*p* = 0.026, β=-0.001), mucosal ridging (*p* = 0.015, β=-1.608), PHQ-9 (*p* = 0.038, β = 0.196), and pain on mouth opening (*p* = 0.038, β = 1.248) were variables significantly associated with high levels of pain and disability. With the equation using the variables, the classification accuracy was estimated to be 84.5% and Nagelkerke R-squared value was 0.258 (*p* = 0.007).

**Table 6:** Logistic regression analysis of physical activity and sleep indices associated with disability level

Variable	Standardized β	Standard error	95% CI	P-value
Time of light physical activity	−0.001	0.000	0.999-1.000	0.026*
Age	−0.035	0.021	0.926–1.007	0.100
Mucosal ridging	−1.608	0.664	0.055–0.735	0.015*
GAD-7	−0.197	0.103	0.671–1.005	0.056
PHQ-9	0.196	0.094	1.011–1.463	0.038*
Pain on mouth opening	1.248	0.603	1.069–11.357	0.038*
Constant	2.227	1.543	-	0.149

Results were obtained from logistic regression analysis

GAD-7: General anxiety disorder-7, PHQ-9: Patient health questionnaire-9

^*^Significant difference: *p* < 0.05

### Amount of physical activity predictive of high disability TMD

**Fig. 3:**
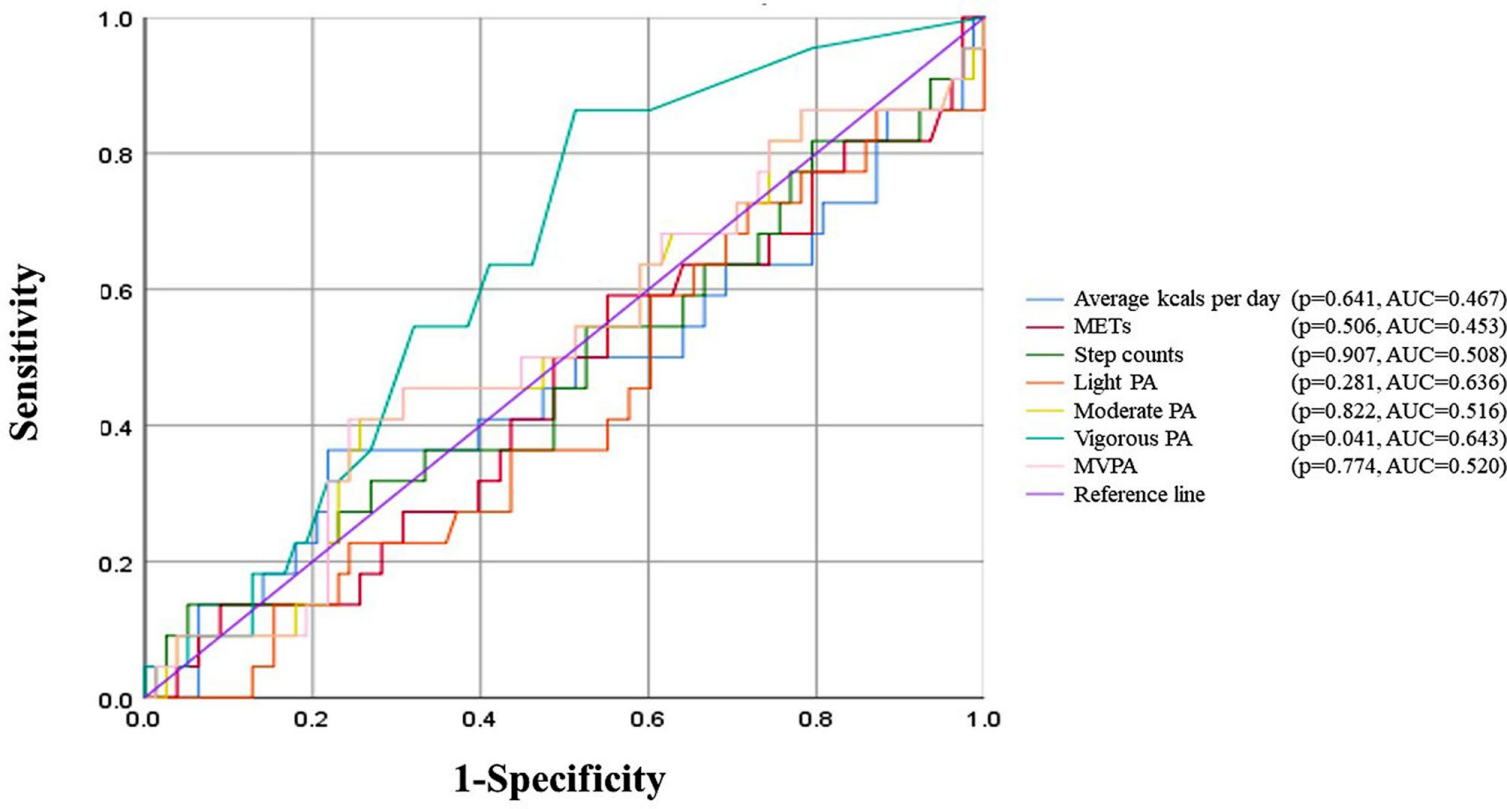
Receiver operating characteristic (ROC) curve. METs, metabolic equivalent of tasks; PA, physical activity; MVPA, moderate-to-vigorous physical activity; AUC, area under the curve

As shown in Fig. 3 and Table 7, the time spent in vigorous physical activity had significant predictive power (*p* = 0.041). The ROC curve analysis shows that vigorous physical activity with a cutoff value of 2.5 min per week leads to an AUC of 0.643 for high disability due to TMD. By using the cutoff value, the patients in this study were classified with sensitivity, specificity, and accuracy of 86.4%, 48.7%, 57.0% respectively.

**Table 7:** Receiver operating characteristics (ROC) curve analyses for evaluation scores to assess influence on disability from Temporomandibular disorders (TMD)

	AUC (95% CI)	Cut off value	Sensitivity	Specificity	PPV	NPV	LR+	LR-	Error rate (%)	P value
Average kcals per day	0.467 (0.318–0.617)	1511.446	0.364	0.782	0.320	0.813	1.670	0.813	31.00	0.641
METs	0.453 (0.315–0.592)	2.441	0.136	0.718	0.120	0.747	0.482	1.203	41.00	0.506
Step counts	0.508 (0.370–0.646)	6.900	0.727	0.397	0.640	0.920	1.206	0.688	15.00	0.907
Light PA	0.636 (0.442–0.708)	3770.000	0.636	0.551	0.560	0.893	1.416	0.661	31.00	0.281
Moderate PA	0.516 (0.375–0.656)	1409.000	0.409	0.744	0.360	0.827	1.598	0.794	29.00	0.822
Vigorous PA	0.643 (0.526–0.761)	2.500	0.864	0.487	0.760	0.960	1.684	0.279	9.00	0.041*
MVPA	0.520 (0.379–0.661)	1447.500	0.409	0.756	0.360	0.827	1.676	0.782	29.00	0.774

AUC, area under the curve; CI, confidence interval; PPV, positive predictive value; NPV, negative predictive value; LR+, positive likelihood ratio; LR-, negative likelihood ratio; METs, metabolic equivalents of task; PA, physical activity; MVPA, moderate to vigorous physical activity

Sensitivity was obtained from TP/(TP + FN) × 100, Specificity was obtained from TN/(TN + FP) × 100, PPV was obtained from TP/(TP + FP) × 100, NPV was obtained from TN/(TN + FN) × 100, Error rate was obtained from (FN + FP)/(TN + TP + FN + FP)

^*^Significant difference, *p* < 0.05

## Discussion

This study is the first attempt to elucidate the relationship between objective levels of physical activity and clinical symptoms in a well-defined group of TMD patients based on DC/TMD. The results of this study showed that moderate to vigorous levels of physical activity was associated with high disability TMD accompanied by higher pain intensity and widespread pain. Such results are in line with a recent study based on a large-scale national database in South Korea reporting that moderate-intensity physical activity was associated with more pain in those with TMD symptoms [51]. Unfortunately, direct comparison of results is limited due to the fact that diagnostic criteria used in the previous study was different as it followed the WHO criteria generally applied in oral health surveys, which examines requires only one item of TMD sign and symptom for diagnosis. Also, only subjective information from questionnaires was gathered to assess physical activity levels. On the other hand, another study based on 8,685 Finnish conscripts reported that those who exercised less frequently showed more TMD symptoms. The evaluation of TMD symptoms was based on 6 self-reporting questions and physical activity level was also assessed with 2 questions [52]. As far as the authors are aware of these studies are the only investigations that have analyzed the relationship between general physical activity level and TMD symptoms but, interpretation of results is restricted due to the subjective nature of gathered data and ambiguity in defining TMD. Therefore, this study based on a TMD patient group defined through a standardized diagnostic process by a calibrated orofacial pain specialist and objectively measured physical activity data holds significance as the results have higher generalizability and reproducibility. The contradictory results from the studies may have originated from the variation in assessment approaches, however aspects of physical activity such as intensity and frequency were handled differently according to the study which may partly explain the discrepant conclusions. Findings based on other musculoskeletal diseases also report inconsistent results. According to the recommendation of the European League Against Rheumatism, physical activity can be beneficial on pain, function, and quality of life to people with rheumatic and musculoskeletal diseases especially, those with osteoarthritis and axial spondyloarthritis suggesting that physical inactivity should be avoided [53, 54]. Another umbrella review also concluded that exercise can reduce pain from fibromyalgia by analyzing thirty-seven recent systematic reviews. On the other hand, an earlier review reported that certain exercise types do help improve function in fibromyalgia patients but some experience an increase in symptoms with higher than moderate intensity exercise that eventually led to incompliance to their exercise regimen [5]. For low back pain, a systematic review and meta-analysis also showed that certain studies comparing high with low or moderate activity reported an elevation in pain level with high-intensity exercise [7]. Such discrepancies may result from the difference in study population, lack of controlling confounding factors, and application of different cut-off levels to separate low, moderate, and high intensity physical activity, all aspects to be considered in future studies to accumulate data that may support the establishment of a standardized physical activity protocol for a certain disease population.

According to the WHO recommendation on physical activity announced in 2020, 150 to 300 min of moderate intensity or 75 to 150 min of vigorous intensity per week, or an equivalent combination of moderate-to-vigorous physical activity can provide many health benefits for both normal and diseased populations [42]. An interesting point is that the past WHO recommendation of 2010 only suggested the minimum amount of activities required for general health improvement. This may reflect the recent realization that more physical activity does not always guarantee better health outcomes and excessive amounts of physical activity may even be harmful in specific situations [55]. Regarding TMD, a recent review paper investigating the effect of competitive sports in this context implied that the incidence of TMD appeared to be increased in athletes compared to non-athletes suggesting an association with vigorous levels of physical activity [56]. The results of our study also support the need of specific guidelines including both the minimum and maximum amount of physical activity for optimal prognosis in TMD patients. It appears that it is crucial to implement exercise only up to the point where symptoms do not exacerbate. ROC analysis of our data showed that as little as 2.5 min/week of vigorous activity was associated with high disability TMD. The equivalent of vigorous activity would be jogging, running, carrying heavy loads upstairs [57].

Based on the obtained logistic regression model, one could easily correlate high pain level and interference of daily life in TMD patients with less light-intensity physical activity. Also, depressive symptoms evaluated with PHQ-9 showed significant correlation with high disability TMD. Such clinical indices could be easily applied in a clinical setting to discern TMD patients with worse symptoms and more comorbidities.

Another point to consider is that objective sleep data was collected in this study. People with poor general health and psychologic problems are more likely to report discrepant subjective sleep information [58]. Although statistically different results could not be found, total sleep time and minutes in bed were both longer and sleep efficiency was higher in the high pain disability group. This does not fall in line with the prevalent knowledge that sufficient sleep time is associated with less TMD pain [59]. Patients in the high disability group got in bed 51 min earlier in average, which was 11:27 PM. This is also contradictory to results from a recent systematic review revealing that later sleep timing was generally associated with worse health outcomes [60]. Some studies do imply that excessive sleep is detrimental to health [61]. The findings of our study place emphasis on a previous report that showed only self-report sleep questionnaire but not actigraphy-measured results were able to differentiate those with TMD from health controls [62].

The relationship between high intensity physical activity and high disability TMD may be mediated by elevated systemic inflammation accompanied by high intensity activities [63]. During high-intensity exercise, skeletal muscles secrete interleukin-6, which are known to influence platelet activation, lymphocyte modulation, and neutrophil function in the dynamic interplay of such blood cells during inflammatory responses [62, 64–66]. Such an increased state of systemic inflammation may directly aggravate TMD related pain however, specific evidence quantitatively linking specific levels of physical activity, inflammation level, and TMD are yet to come. The inflammatory markers investigated in this study were ratio values selected based on previous studies showing there higher accuracy in reflecting systemic inflammatory status and long-term disease prognosis compared to absolute values [67, 68]. All investigated hematological inflammatory indices showed a trend of increased inflammation however, the difference between groups was not statistically significant. Systemic inflammation is generally known to be associated with sleep disturbance and obesity [69, 70]. The lack of distinction between groups may have arisen from the absence of distinct differences in causal factors, such as deprivation of sleep and BMI. Future studies based on different grouping criteria are necessary to further investigate the correlation among physical activity, sleep, pain, and inflammation.

There are some limitations of this study to consider. Due to the cross-sectional design of this study, the causal relationship between physical activity level and TMD cannot be derived. Therefore, experimental and longitudinally designed studies are needed to confirm the findings. Second, despite applying a validated accelerometer for physical activity evaluation, measurements have limitations in reflecting the true amount of physical activity. For instance, basal metabolic rate is not taken into account and resistance exercise or leg exercise are prone to underestimation [71]. Objective measurements alone are not consistently related to results from questionnaires, so adopting both subject and object investigations would be more effective [72]. Third, there is no consensus on cut-off values of vector magnitude for classifying physical activity intensity with a triaxial accelerometer worn on the wrist. A calibration study has been conducted only recently [50]. This study used cut-offs from the calibration study instead of using values derived from studies with devices worn on the hip for more accurate classification. Fourth, the shortcomings of actigraphy measured sleep data compared to polysomnography should be considered in interpreting results. Furthermore, sleep data was not supported by sleep dairy logging. Lastly, potential biases may exist due to the sequential recruitment of patients. Subject recruitment occurred over an 8-month period and seasonal effects on psychological condition and physical activity may be present. Also those who were excluded from the study may have an inherent characteristic that is essential for investigation of the study subject. Future studies should consider recruiting subjects considering such aspects [71, 73–76]. In the present study, the association between TMD and everyday physical activity was investigated. However, it is noteworthy that certain types of exercises may aggravate or alleviate TMD [56] and additional studies are called upon to assess the effect of certain sports activities based on objective measurements of intensity and frequency to be able to provide recommendations to the patients.

## Conclusions

TMD patients showed a clear difference in terms of physical activity according to disability level. Both moderate- and high-intensity physical activity was positively associated with high disability. This study showed that the time of vigorous activity associated with high disability TMD was 2.5 min per week. Based on such findings, TMD patients are recommended to minimize vigorous activities while engaging in more light-intensity physical activity to avoid symptom aggravation. Clinicians should objectively evaluate general physical activity level in TMD patients and be able to provide recommendations for better treatment outcomes. Further investigations are necessary to provide more detailed guidelines regarding the optimum intensity and frequency of physical activity for TMD patients.

## Data Availability

The datasets generated and/or analysed during the current study are not publicly available due ethical reasons but are available from the corresponding author on reasonable request.

## Abbreviations

TMD: temporomandibular disorders
DC/TMD: diagnostic criteria for TMD
IPAQ: international physical activity questionnaire
TSK-TMD: Tampa scale of kinesiophobia for TMD
SF-36: Short form-36
COMPASS 31: composite autonomic symptom score 31
MPQ: McGill pain questionnaire
PSQI: Pittsburgh sleep quality index
ESS: Epworth sleepiness scale
FAI: fatigue assessment instrument
BDI: Beck depression index
BAI: Beck anxiety index
CSI: central sensitization index
PILL: Pennebaker index of limbic languidness
PSS: perceived stress scale
SS: symptom severity scale
WPI: widespread pain index
FIQ: fibromyalgia impact questionnaire
JFLS-20: jaw function limitation scale-20
OBC: oral behavior checklist
GAD-7: general anxiety disorder-7
PHQ-9: patient health questionnaire-9
PHQ-15: patient health questionnaire-15
CBC: complete blood cell count
ESR: erythrocyte sedimentation rate
CRP: C-reactive protein
RF: rheumatoid factor
FANA: fluorescent antinuclear antibody
anti-CCP: anti-cyclic citrullinated peptide
PLR: platelet-to-lymphocyte ratio
NLR: neutrophil-lymphocyte ratio
dNLR: derived neutrophil-lymphocyte ratio
LMR: lymphocyte-monocyte ratio
SII: systemic inflammatory index
GCPS: graded chronic pain scale
